# THEORIZING FORTITUDE, FRIENDSHIP, AND OLD AGE IN LORE SEGAL’S SHORT STORY CYCLE “LADIES LUNCH”

**DOI:** 10.1093/geroni/igae098.1984

**Published:** 2024-12-31

**Authors:** Eva-Maria Trinkaus

**Affiliations:** University of Klagenfurt, Austria, Klagenfurt, Karnten, Austria

## Abstract

This contribution addresses the question of ‘fortitude’ from a literary studies perspective. In my narrative analysis of Lore Segal’s short story cycle “Ladies Lunch” (2023) I look at the representation of fortitude in the context of friendship in old age. Fictional narratives about female friendships in old age, Chivers argues, can be part of “constructive narratives of aging” in an “imaginary world that can reflect and especially affect circulating social thought” (2003). Friendship in old age, in Segal’s fictional text, constitutes a form of care among the women who partake in their regular lunch meetings in which they contest the readers’ views on stereotypes of old age, but at the same time, also express personal vulnerabilities in opposition to a “decline narrative” (Gulette 2001). On the contrary, the kind of reciprocal care they experience comes from a vulnerability that manifests as a “radical openness towards surprising possibilities” (Hirsch 2016). In theorizing fortitude, this narrative analysis addresses the ways fortitude manifests in ways that do not necessarily address danger or pain in the literal sense of the word. Rather, it provides a perspective that juxtaposes fortitude and age in order to address courage in accepting situations and the creative adaptation to those within the story.

